# From guidelines to radiology practice: navigating the 2023 ASCO guidelines for advanced gastroesophageal cancer and beyond

**DOI:** 10.1007/s00261-024-04499-y

**Published:** 2024-08-09

**Authors:** Charit Tippareddy, Orlando M. Martinez, Andrew R. Benza, Kaustav Bera, Nikhil Ramaiya, Sree Harsha Tirumani

**Affiliations:** 1 Department of Radiology, University Hospitals Cleveland Medical Center, Case Western Reserve University, 1110 Euclid Ave, Cleveland, OH 44106 USA; 2 Case Western Reserve University School of Medicine, Cleveland, OH USA

**Keywords:** Gastroesophageal junction, Medical oncology, Review, Immunotherapy, Radiotherapy, Precision medicine

## Abstract

The American Society of Clinical Oncology (ASCO) updated the guidelines for the treatment of advanced gastroesophageal (GE) cancer in 2023, signifying a major shift towards targeted therapeutics and precision medicine. This article serves as an imaging-based review of recent developments in the care of patients with GE cancer. We cover the epidemiology, the developing treatment paradigms, and the imaging assessment of GE malignancy. In addition, this review aims to familiarize radiologists with the unique adverse effects pertaining to therapeutics, surgeries, radiation therapies, and associated imaging corollaries. A case-based approach will be used to both explore the efficacy of modern treatments and demonstrate their adverse effects, such as chemotherapy-associated pneumonitis, radiation esophagitis, and anastomotic failure. With this comprehensive exploration of gastroesophageal cancer, radiologists will be equipped with the essential tools to inform the treatment decisions made by medical oncologists, radiation oncologists, and surgical oncologists in the new era of precision medicine.

**Graphical abstract:**

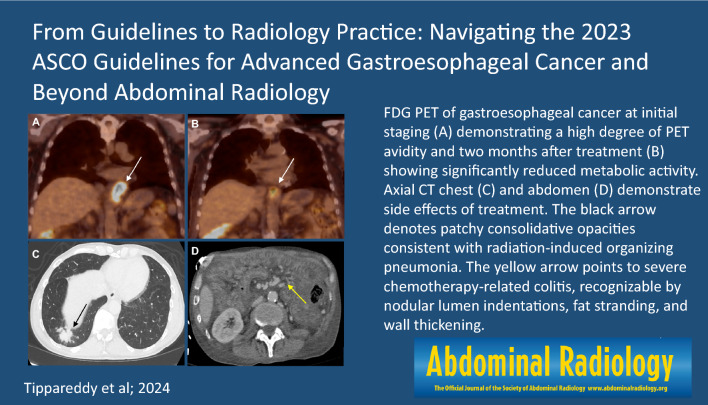

## Introduction

More than 1.6 million new diagnoses of gastroesophageal (GE) cancer were made globally in 2020, and in the same year, over 1.3 million deaths were attributed to the malignancy. These figures made gastroesophageal cancer the second and third-highest-ranked cancer internationally in terms of mortality and incidence, respectively [1]. In 2022, the United States alone saw approximately 47,000 new cases and 27,000 deaths due to gastroesophageal cancer [1]. The grave nature of GE cancer has inspired numerous surgical interventions, such as partial gastrectomy (which was first accomplished in 1881 by Theodor Billroth), total gastrectomy, gastroduodenostomy, gastrojejunostomy, esophagectomy, and esophagogastrostomy. There has been similar progress in the development of therapeutic agents, including traditional chemotherapies, anti-angiogenic, immune checkpoint inhibitors (ICIs), tyrosine kinase inhibitors (TKIs), monoclonal antibodies, and HER2 inhibitors. The combination of these treatments with advancing radiation therapies has significantly enhanced the overall management of gastroesophageal cancer, leading to improved survival rates and an increase in the 5-year life expectancy for patients [2, 3].

Care for these patients is complex and intensive, requiring a multidisciplinary team. With the advent of new and varied treatment options, radiologists must possess a foundational understanding of disease management to effectively identify and interpret both the positive treatment responses and the adverse events associated with each therapeutic. Thus, this article illuminates the power of diagnostic imaging in the evolving landscape of gastroesophageal cancer management, equipping radiologists with the tools necessary to contribute to the multidisciplinary care team more effectively.

## Gastroesophageal cancer: epidemiology, risk factors, and pathogenesis

Although significant progress has been made in the treatment of gastroesophageal malignancies over the past decade, gastroesophageal cancer still has the second-highest mortality of all cancers [4]. GE cancer accounts for 2.5% of recent cancer diagnoses in the United States, with the lifetime risk of developing GE cancer at an estimated 1/56 for men and 1/114 for women [4]. According to the American Cancer Society's 2024 projections, there are expected to be 26,890 new cases and 10,880 fatalities from gastric cancer, in addition to 22,370 new cases and 16,130 deaths from esophageal cancer in the U.S. alone [5].

When caught early, GE cancers will present in or around the gastroesophageal junction, universally recognized as the region where the esophagus and lower esophageal sphincter meet the upper stomach. Unfortunately, these malignancies are often metastatic on presentation, with approximately 30% having distant metastasis and another 30% demonstrating local spread on initial staging scan [4, 6]. Consequently, the average five-year survival rate for GE cancer is just 22%, though this is a significant improvement from the low rate of 5% in the 1960s and 1970s [3–7]. The risk of developing gastroesophageal cancer is influenced by various factors, including lifestyle, socioeconomic status, prior history of gastric reflux, environmental exposures, micronutrient deficiencies, and age/gender differences [7]. Inherited mutations, notably in the RHBDF2 gene or those linked with Bloom syndrome, significantly contribute to one's risk of developing GE malignancy [8]. Other acquired mutations associated with esophageal cancer include TP53, NOTCH, and MTOR, as well as amplification of the following genes: AKT2, EGFR, ERBB2 (HER2), FGFR1, KRAS, MDM2, and PIK3CA [9]. Altered expression of these genes during periods of cellular stress can dysregulate the cell cycle and contribute to a stepwise progression of cancer [8, 9].

There are two main types of primary esophageal cancers: squamous cell carcinoma and adenocarcinoma. Squamous cell carcinoma is the predominant GE neoplasm globally (85%) and is associated with factors like smoking and alcohol consumption. In contrast, adenocarcinoma is less prevalent internationally (14%) and typically occurs in individuals with Barrett’s esophagus [7, 8]. Interestingly, the distribution is reversed in the United States and other Western countries, with esophageal adenocarcinoma accounting for 80% of cases. This disparity is attributed to the rising rates of gastroesophageal reflux disease (GERD) and obesity, factors more prevalent in the West [10]. Microscopically, well-differentiated squamous cell carcinomas can be described by the presence of immunohistochemical markers, such as CK5/6 or p63, and by features such as keratin pearls, cell keratinization, and intercellular bridges [10]. Adenocarcinoma, in contrast, is histologically characterized by glandular differentiation that may present variably, including tubular, tubulopapillary, or papillary structures, with a small subset presenting mucinous differentiation [8, 10]. Genetic analysis of an adenocarcinoma may reveal that the cells have acquired mutations in genes such as p16 and p53 [11]. Meanwhile, up to 95% of gastric cancers are adenocarcinomas of the intestinal or diffuse type, with a small percentage of gastrointestinal stromal tumors (GISTs), neuroendocrine tumors, or lymphomas [12]. Like esophageal adenocarcinomas, gastric adenocarcinomas are histologically characterized by glandular differentiation [12]. In addition to traditional pathology tissue slides, next-generation sequencing (NGS) such as whole genome sequencing, whole exome sequencing, and RNA sequencing have become pivotal in understanding cancer pathogenesis [13]. NGS has become ubiquitous and is integral in the management of various malignancies, including gastroesophageal cancer. It has become the standard of care and is explicitly reimbursed by insurance companies in the United States [14]. With NGS, the number of potential biomarker targets has rapidly evolved to include HER2, PD-L1, EGFR, PARP, FGFR-2, and more, with a number of active clinical trials underway [13].

**Fig. 1:**
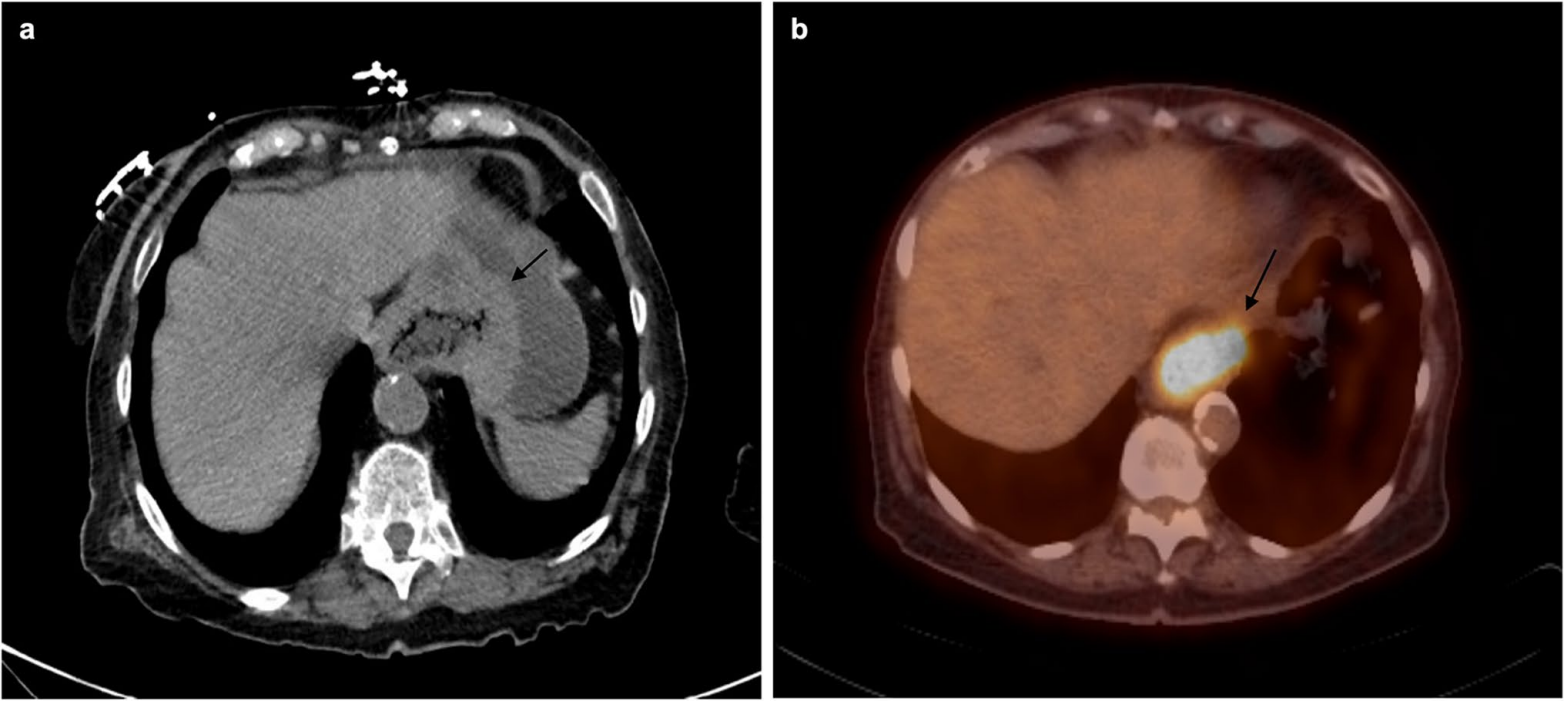
Axial CT scan (**a**) demonstrating an infiltrative circumferential mass in the distal esophagus extending into the proximal stomach (arrow) that disrupts the normal smooth curvature of the stomach. The following steps include confirmation and further evaluation via endoscopy. PET/CT F-fluorodeoxyglucose (**b**) avidity associated with lower esophageal wall thickening extending into the GE junction and gastric cardia (arrow) corresponding to known GE malignancy

The gold standard for diagnosing esophageal cancer is upper endoscopy combined with biopsy and histopathological analysis; endoscopic ultrasound (EUS) is able to accurately differentiate between the esophageal layers and assess tumor invasion depth, demonstrating a 76–89% accuracy for T staging compared to 49–59% for CT [15]. In N staging, the accuracy of EUS ranges from 72 to 80% compared to 46–58% for CT [15]. The actual utility of radiologists begins with M staging. CT is the most common modality for initial staging, quickly followed by FDG PET (Fig. 1). While FDG PET has been shown to be more sensitive, a lack of anatomical detail and scanner unavailability have limited its use. With the advent of fusion PET/CT, this has quickly become a mainstay of initial staging (Table 1) [15].

**Table 1:** National Comprehensive Cancer Network guidelines for grading gastroesophageal cancer based on clinical and pathologic findings. Version 2.2024, April 23, 2024

Grading criteria	Description
Tumor size	T1: Tumor invades lamina propria, muscularis mucosae, or submucosa
	T2: Tumor invades muscularis propria or subserosa
	T3: Tumor invades the adventitia
	T4: Tumor invades esophageal wall or peri-esophageal structures (i.e. pleura, pericardium, trachea)
	Tis: Tumor in situ, or cancer cells only growing at the cell layer where they began
	TX: There is no information about the primary tumor, or it can’t be measured
Lymph node involvement	N0: No regional lymph node metastasis
	N1: Metastasis in 1 to 2 regional lymph nodes
	N2: Metastasis in 3 to 6 regional lymph nodes
	N3: Metastasis in 7 or more regional lymph nodes
	NX: Cancer in nearby lymph nodes cannot be measured
Metastasis	M0: No distant metastasis
	M1: Distant metastasis (e.g., liver, lung, bone)
Histological grade	G1: Well-differentiated (low grade)
	G2: Moderately differentiated (intermediate grade)
	G3: Poorly differentiated (high grade)
	GX: Grade cannot be assessed
Location (Siewert-type)	Type I: Proximal to gastroesophageal junction
	Type II: 1 cm above or 2 cm below the gastroesophageal junction
	Type III: 2 to 5 cm below the gastroesophageal junction
Stage	I: T1/T2, N0, M0
	II: T3/T4a, N0, M0
	IIIA: T1/T2, N1, M0; T3, N0, M0
	IIIB: T4a, N1, M0; T4b, N0/N1, M0
	IIIC: Any T, N2/N3, M0
	IV: Any T, Any N, M1

## Updated ASCO guidelines and their implications for treatment

**Fig. 2:**
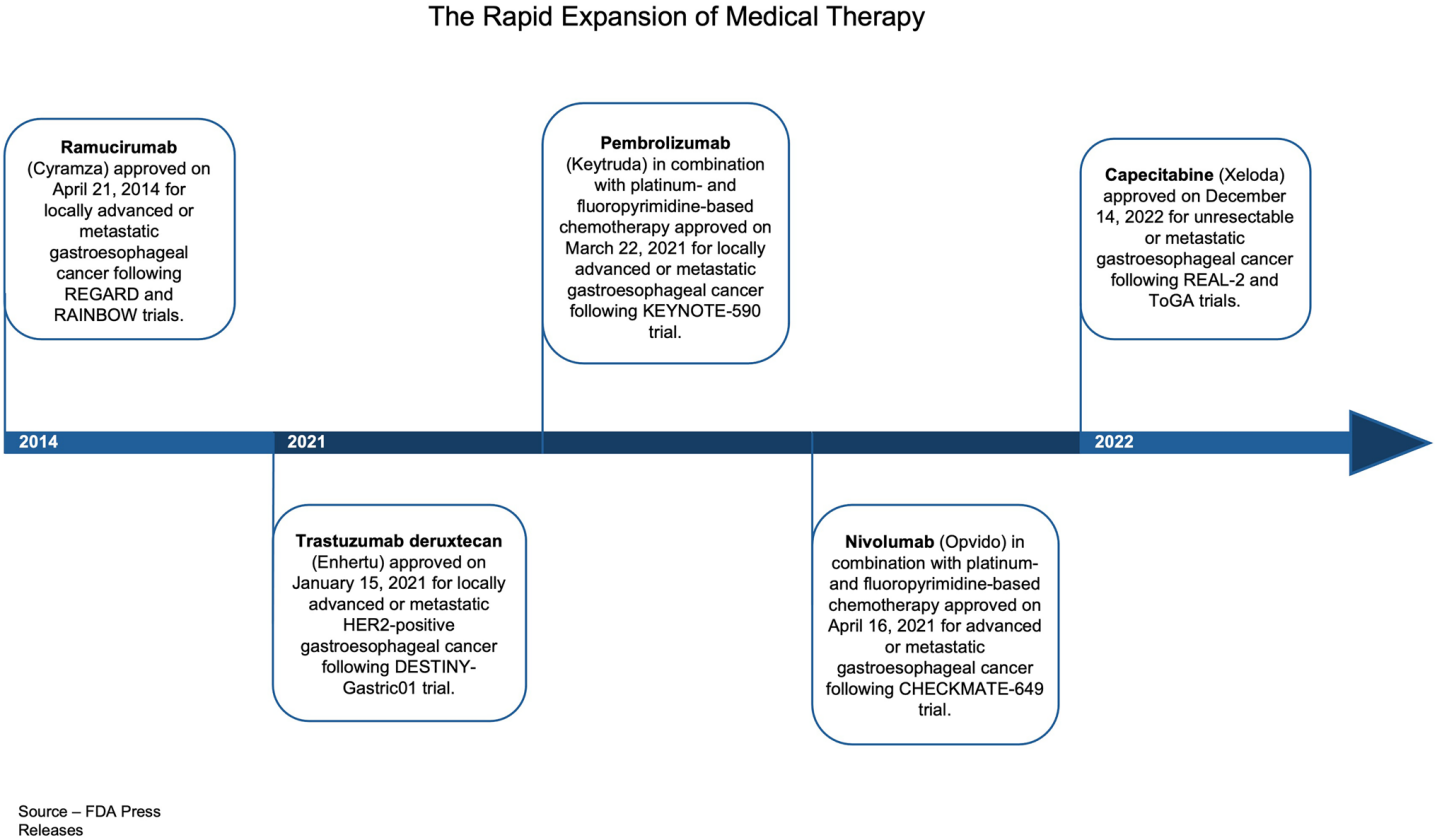
Timeline demonstrating the progression of targeted therapies once used for other malignancies that have recently gained approval for GE cancer

**Fig. 3:**
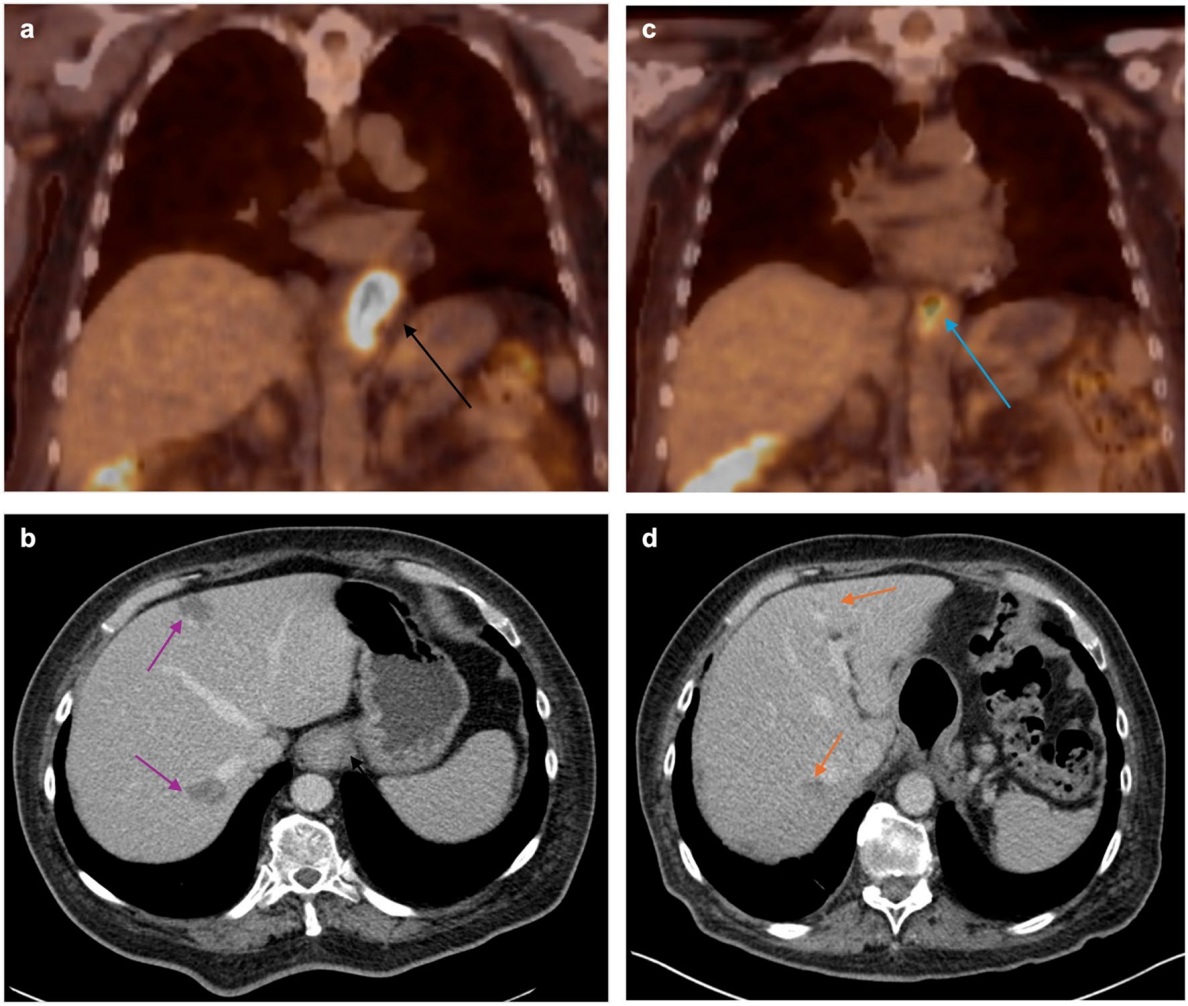
FDG PET/CT of a distal esophageal adenocarcinoma at the time of initial staging (**a**) demonstrating a high degree of PET avidity with a max SUV of 12.7 in addition to a CT (**b**) showing a circumferential mass of the distal esophagus (black arrow) and hepatic metastases (purple arrows). FDG PET/CT after 2 months (**c**) of chemoradiation with Taxol and carboplatin showing significantly reduced metabolic activity with a max SUV of 4.2 (blue arrow). Three and a half years after esophagectomy and initiation of nivolumab, CT (**d**) shows treated hepatic metastases (orange arrows) without evidence of active disease

The updated guidelines highlight the rapid development of novel treatment strategies for advanced gastroesophageal cancer since the American Society of Clinical Oncology (ASCO) last issued recommendations [2]. Pharmacology has explicitly evolved dramatically, transitioning from broadly prescribed 5-FU chemotherapy, first approved by the FDA in 1962, to more targeted therapies introduced in the twenty-first century (Fig. 2). For instance, therapeutics that target an individual patient’s tumor phenotype, like ramucirumab and pembrolizumab, received FDA approval for use in GE cancer in 2014 and 2021, respectively. Furthermore, the industry found that anti-neoplastic agents that were previously approved for other malignancies, such as trastuzumab for breast cancer, could be repurposed for care in GE cancer patients [1]. As of 2021, trastuzumab holds expanded approval for use in gastroesophageal cancer, colorectal cancer, and non-small cell lung cancer [16]. Capecitabine was also given updated labeling in 2022 and has since been incorporated into the treatment algorithm for advanced GE cancer. These improved treatment approaches have revolutionized the management of local gastroesophageal cancer and ushered in an age of personalized medicine (Fig. 3).

To recognize this advancement, the American Society of Clinical Oncology updated its guidelines in 2023 for treating advanced gastroesophageal cancer, incorporating groundbreaking treatment modalities into the treatment paradigm. The implication of these guidelines is best realized after understanding the critical terms that shape the treatment algorithm. The guidelines frequently utilize the term “advanced gastroesophageal cancer,” or malignancies that have significantly progressed, becoming invasive and spreading beyond the original site, often making them inoperable. One specific type of tumor discussed in the update is one that over-expresses the human epidermal growth factor receptor 2 (HER2) receptor, a common breast cancer marker that similarly portends an aggressive GE tumor by manipulating cell growth and replication. Despite their biochemical complexity, the ASCO guidelines emphasize that HER2-positive tumors show a robust response to specific treatment regimens [17]. Trastuzumab-based chemotherapies, for example, have drastically improved the prognosis of advanced HER2-positive gastric cancer, prolonging median overall survival by nearly 20% [17]. Similarly, programmed death-ligand 1 (PD-L1), a biomarker abnormally expressed in malignant cells, has also become crucial in determining the effectiveness of specific immunotherapies [16, 18]. To aid immunotherapy selection, the 2023 ASCO update introduced the Tumor Proportion Score (TPS) and the Combined Positive Score (CPS), which evaluate the percentage of tumor cells, lymphocytes, and macrophages that express PD-L1 and the relative susceptibilities of different treatment options [16, 18].

**Fig. 4:**
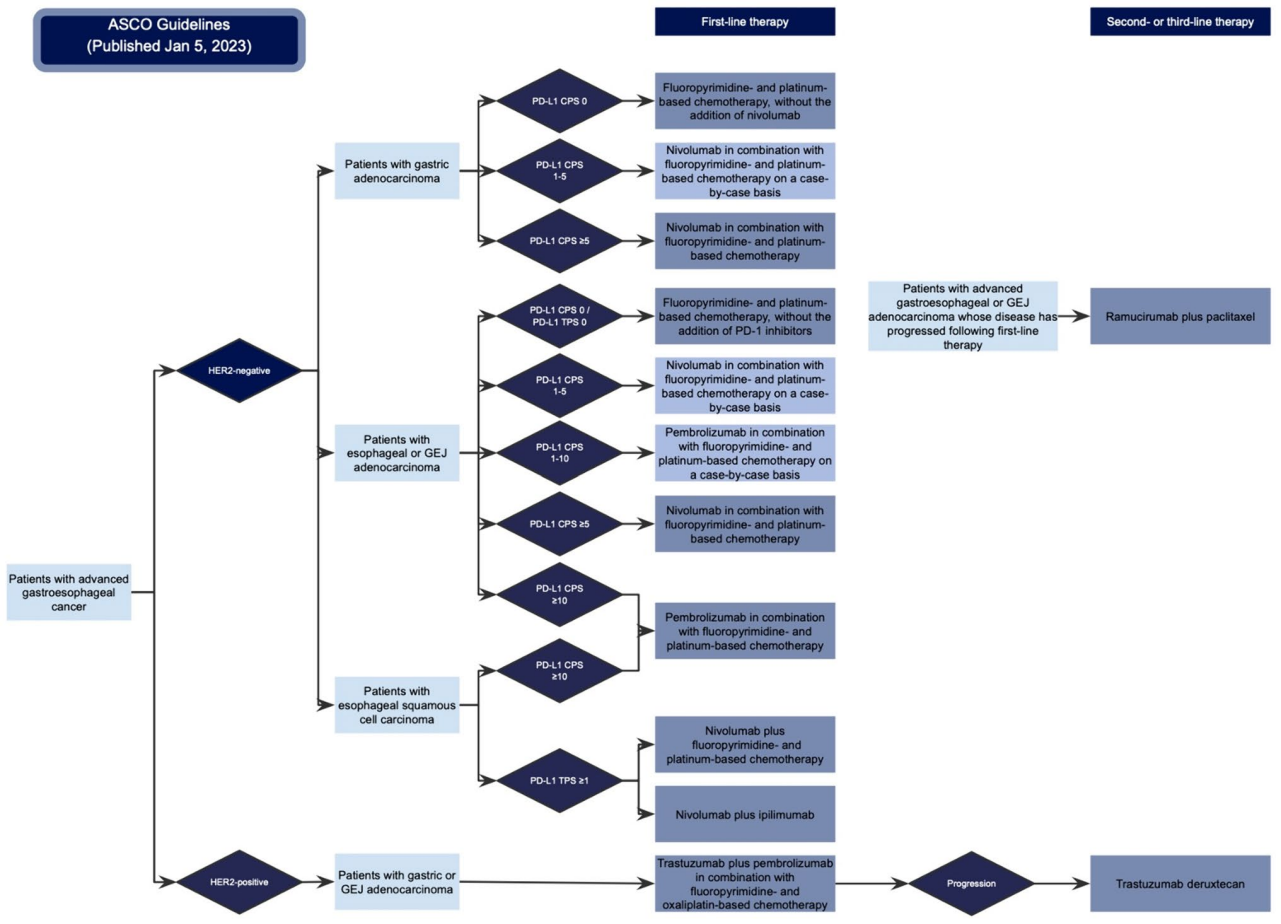
Algorithm demonstrating the newly released treatment recommendations by the American Society of Clinical Oncology (ASCO) in 2023. *TPS* tumor proportion score; *CPS* combined positive score

The treatment algorithm presented in the ASCO 2023 guidelines denotes a monumental change in the approach to treating esophageal cancer (Fig. 4). Supported by compelling evidence from pivotal clinical trials such as KEYNOTE-590, CHECKMATE-648, and ESCORT-1st, the 2023 recommendations shine a spotlight on immunotherapy as a complementary option alongside chemotherapy for recurrent or metastatic squamous cell carcinoma [19–21]. The nuanced strategy proposed is driven by various factors such as HER2 status, PD-L1 expression levels (both TPS and CPS), and the specific nature of the tumor, whether esophageal squamous cell carcinoma or adenocarcinoma [22]. For instance, in HER2-negative gastric adenocarcinomas with a PD-L1 CPS of ≥ 5, the updated first-line therapy recommendation is a combination of nivolumab and chemotherapy [18]. Similarly, the same combination therapy is advised for HER2-negative esophageal or gastroesophageal junction adenocarcinomas with a PD-L1 CPS of ≥ 5 [23]. Lastly, the update underscores the utility of pembrolizumab in combination with chemotherapy for patients with esophageal or gastroesophageal junction adenocarcinomas that are HER2-negative and have a PD-L1 CPS of ≥ 10 [18].

Now, loosely resembling its twentieth-century origins, the management of esophageal cancer has evolved to achieve the precision-driven methods of today, as outlined by the 2023 ASCO guidelines.

## Imaging-based assessment of treatment adverse effects

The side effects of anti-neoplastic treatments have long been a concern of patients and clinicians alike. While the adverse effects of past treatments were mainly monitored clinically, many contemporary interventions have side effects that produce early subclinical imaging findings. As a result, frequent imaging studies have become crucial in monitoring for both treatment response and toxicities that may require alternative forms of care. Radiologists interpreting these images must be aware of the side effect profiles when evaluating patients undergoing specific treatments, as understanding the common presentations of toxicities will help radiologists differentiate between drug events and disease progression. The following sections will provide the radiographic findings of the most common and disruptive complications of medical chemotherapy and radiotherapy (Table 2). In addition, common postoperative challenges will also be highlighted.

**Table 2:** Classes of chemotherapy and their associated side effects

Chemotherapy classes	Associated adverse effects
Fluoropyrimidine	Pericarditis
	Myocarditis
	Takotsubo cardiomyopathy
	Hepatic steatosis
	Biliary strictures
	Neurotoxicity
Platinum-based chemotherapy	Hepatomegaly
	Ascites
	Pleural effusion
	Gallbladder wall thickening
	Periportal edema
Immune checkpoint inhibitors	Pneumonitis
	Colitis
	Hepatitis
	Pancreatitis
	Thyroiditis
	Hypophysitis
	Arthritis
	Lymphadenopathy
Targeted therapies	Pneumonitis
	Colitis
	Thyroiditis
	PRES
	Pancreatitis
	Cholecystitis

Medications classified as fluoropyrimidines include 5-flurouracil, capecitabine, floxuridine. Agents in the platinum-based chemotherapy group include carboplatin, oxaliplatin, and cisplatin. Immune checkpoint inhibitors are therapies such as nivolumab, pembrolizumab, and ipilimumab. Lastly, the targeted therapies are drugs such as trastuzumab and ramucirumab

## Medication associated adverse effects

### Pulmonary findings

**Fig. 5:**
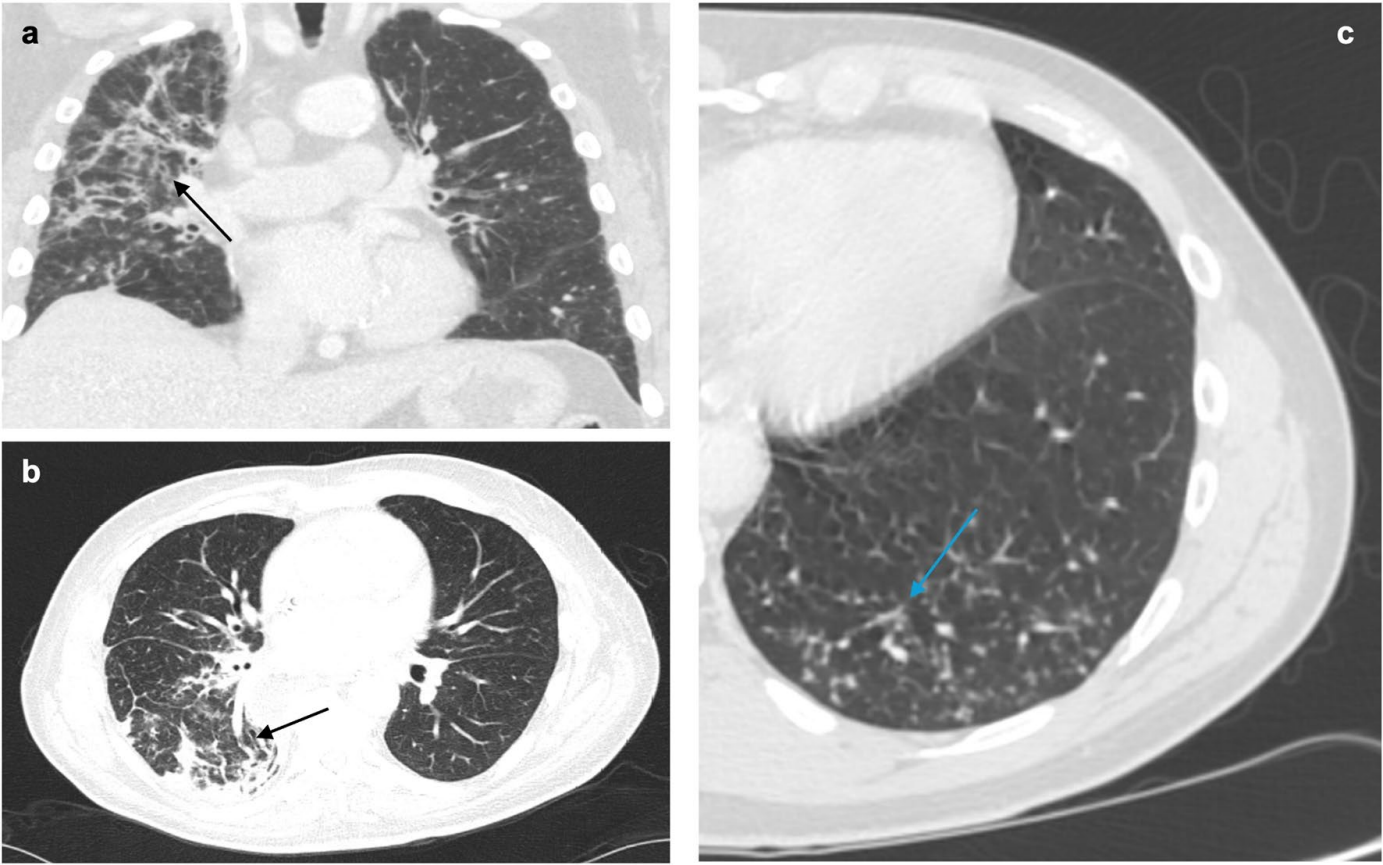
HRCT showing pneumonitis; predominant findings of antineoplastic agent–induced pneumonitis in axial (**a**) and coronal (**b**, **c**) planes are diffuse/multifocal ground-glass opacities (black arrows) or tree-in-bud opacities (blue arrow) with intralobular interstitial thickening and consolidations

Pulmonary injury is a known complication of cancer treatment. In patients with advanced GE cancer, many of the pharmacologic options utilized can inadvertently produce treatment-associated pneumonitis. The incidence is estimated at around 3%–6% of medically managed patients [24]. Pneumonitis was described initially in the KEYNOTE-590 trial, which revealed that patients receiving pembrolizumab, a fluoropyrimidine agent, or platinum-based chemotherapy were more likely to develop pneumonitis [25]. Further work performed under clinical trials CHECKMATE-659, CHECKMATE-648, KEYNOTE-811, and DESTINY uncovered that the immunotherapies nivolumab, ipilimumab, and trastuzumab could also cause pneumonitis in as many as 10% of patients [25, 26]. Drug-associated interstitial pneumonitis and fibrosis result in ground-glass opacities, focal areas of consolidation, and irregular linear opacities that tend to involve the lower zones of the lungs (Fig. 5). These changes are optimally viewed using high-resolution CT imaging (HRCT) and warrant consideration of modifying the therapeutic strategy.

**Fig. 6:**
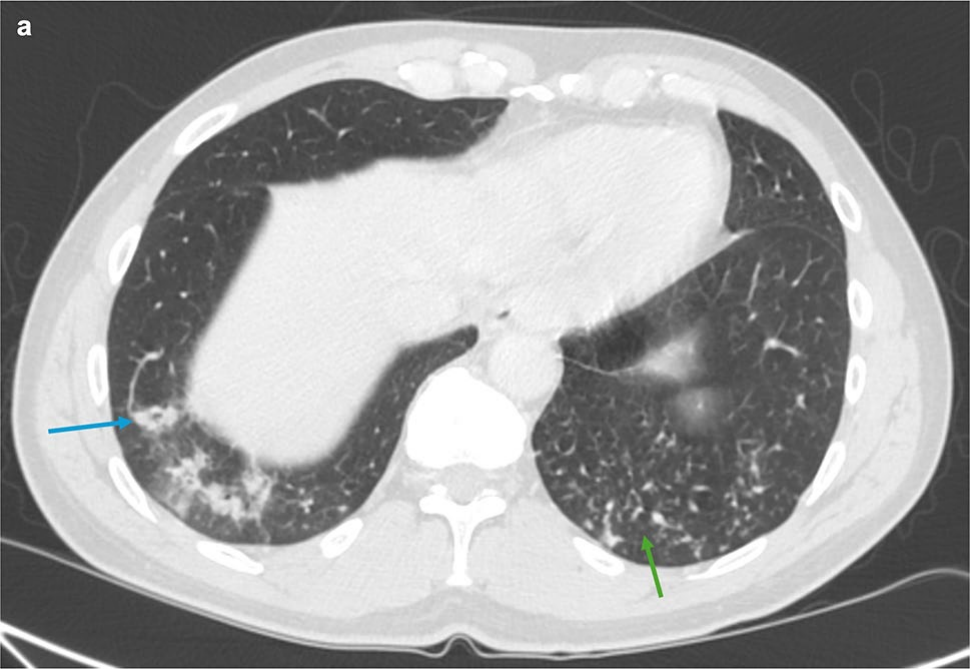
HRCT of the chest (**a**) showing cryptogenic organizing pneumonia, which typically demonstrates the reverse halo sign (blue arrow), nodular tree-in-bud lesions (green arrow), patchy ground-glass opacities, and inflammation of bronchioles and alveoli. These are all suggestive of an organizing pneumonia pattern

There can be other pulmonary manifestations in patients undergoing different types of chemotherapy. For example, patients with advanced GE cancer receiving medical therapy such as nivolumab or the combination chemotherapy FOLFOX—a regimen that includes leucovorin calcium—can also develop cryptogenic organizing pneumonia (COP) [27]. This diffuse interstitial disease deteriorates the distal and respiratory bronchioles, as well as the alveolar walls (Fig. 5) [27]. On HRCT, COP is characterized by patchy consolidation with a predominantly subpleural and peribronchial distribution, small, ill-defined peribronchial or peribronchiolar nodules, and bronchial wall thickening (Fig. 6). Another potential pulmonary side effect is nonspecific interstitial pneumonia (NSIP), a homogeneous interstitial inflammation characterized by infiltration of mononuclear cells and fibrosis [28]. Patients with NSIP uniquely develop reactive hyperplasia of type-2 pneumocytes, and imaging usually reveals predominant changes in the lower parts of the lungs, including reticular opacities, fibrosis, and traction bronchiectasis [28].

Furthermore, diffuse alveolar damage is an adverse effect that should not be mistaken because it can progress to irreversible destruction of the airway architecture. Early findings include homogeneous or inhomogeneous ground glass opacities on radiographs and HRCT, while the late phase is dominated by fibrosis [28]. Finally, eosinophilic pneumonia, characterized by eosinophils and macrophages invading the alveolar septa and thickening the alveolar walls, should also not be disregarded [28].

### Cardiovascular manifestations

**Fig. 7:**
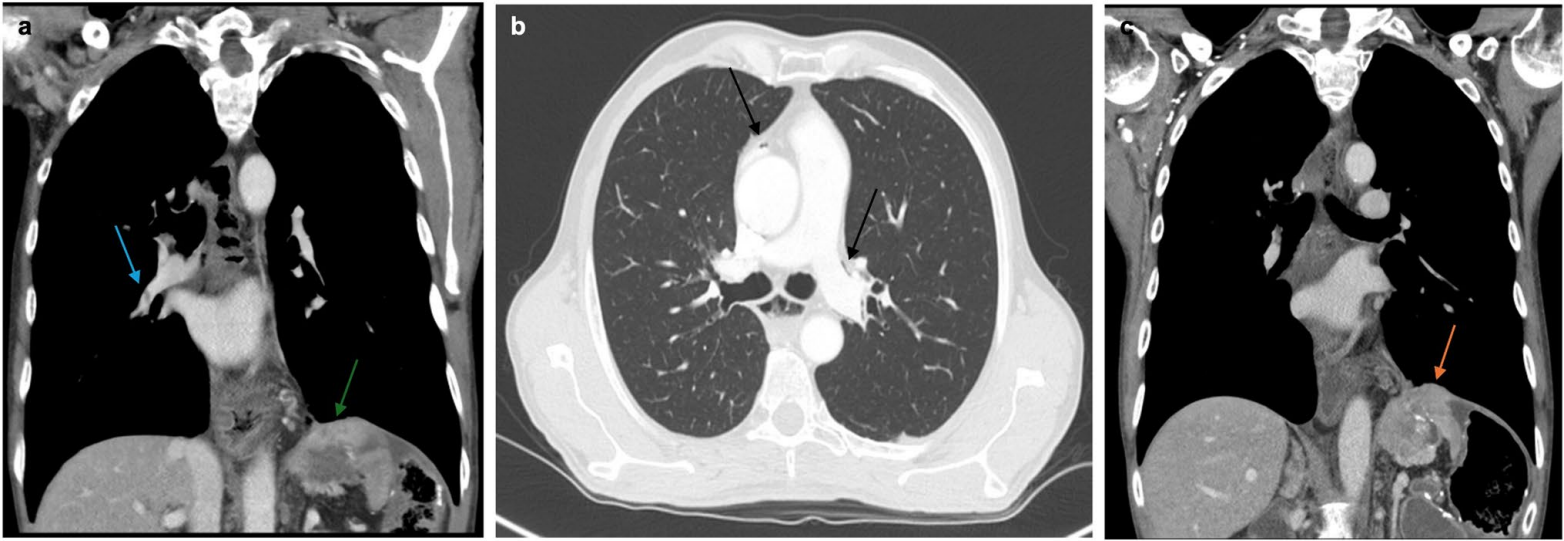
Contrast-enhanced CT scans of the chest after initiation of an anti-angiogenic agent (**a**) demonstrates a right lower lobe pulmonary embolus (blue arrow) with a 32 mm enhancing lesion in the GE junction (green arrow). Around the same time, another CT (**b**) showed pneumomediastinum (black arrows). One month after stopping the anti-angiogenic agent, repeat CT (**c**) showed resolution of the right lower lobe pulmonary embolus and pneumomediastinum with rapid interval growth in the size of the lesion (orange arrow) to 48 mm. Timeline demonstrates the balance between treatment related adverse side effects and treatment of the primary malignancy

Though less common, cardiovascular side effects have also been observed during medical treatment of advanced gastroesophageal cancer. Immune checkpoint inhibitors and fluoropyrimidine agents can be cardio-toxic through dysregulation of the cell cycle checkpoints that are vital for myocardial and endovascular homeostasis [29, 30]. This toxicity can present variably, including pericarditis, myocarditis, cardiomyopathy, vasculitis, and vascular pathologies (Fig. 7). For example, approximately 8.2%-9.4% of patients receiving platinum-based therapies experience vascular thromboembolic events such as pulmonary embolism, venous thrombosis, and accelerated atherosclerosis [31, 32]. Atherosclerotic disease is most prevalent during the initial 6 months of therapy and often found incidentally on restaging scans [31]. Pericardial effusion is seen when using platinum-based chemotherapies, such as carboplatin, oxaliplatin, and cisplatin [29, 30]. These cardiovascular adverse effects are associated with high mortality rates; for example, patients with ICI-associated myocarditis have mortality rates ranging from 25–50% compared to 4% for non-ICI myocarditis [30].

Diagnosis of cardiovascular injury involves long-term follow-ups, regular examinations, and sequential cardiac function tests. Imaging modalities such as echocardiography, PET, and cardiac MRI (CMR) are the mainstay for assessing myocardial function in addition to valvular and pericardial involvement [33]. Despite the widespread utility of echocardiography, CMR is currently considered the gold standard because of its unique applications to characterize the anatomic and functional properties of lesions [34]. For example, a technique such as the late gadolinium enhancement (LGE) technique emphasizes regional differences in myocardial extracellular volume and different uptake and washout to detect pathologies such as thrombus, necrosis, fibrosis, or metastasis [35]. Likewise, the elevated transverse relaxation time (T2) approach for CMR can identify increased water content and free water, allowing it to identify areas of myocardial edema [34, 36]. This technique, called quantitative T2 mapping, has been reliably used to diagnose, stage, and track myocardial injury.

### Hepatobiliary injury

**Fig. 8:**
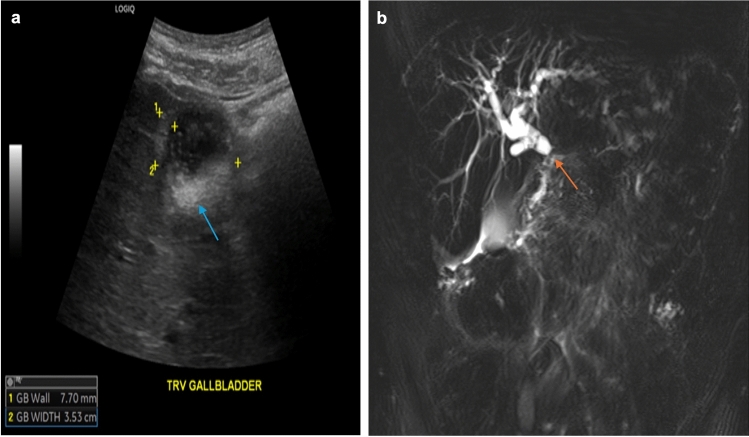
Findings related to fluoropyrimidine and platinum-based chemotherapy toxicity. Ultrasound (**a**) shows gallbladder sludge/stones (blue arrow) and wall thickening (calipers denote wall). MRCP (**b**) shows diffuse intrahepatic biliary ductal dilatation with stricture (orange arrow) in the periportal region

**Fig. 9:**
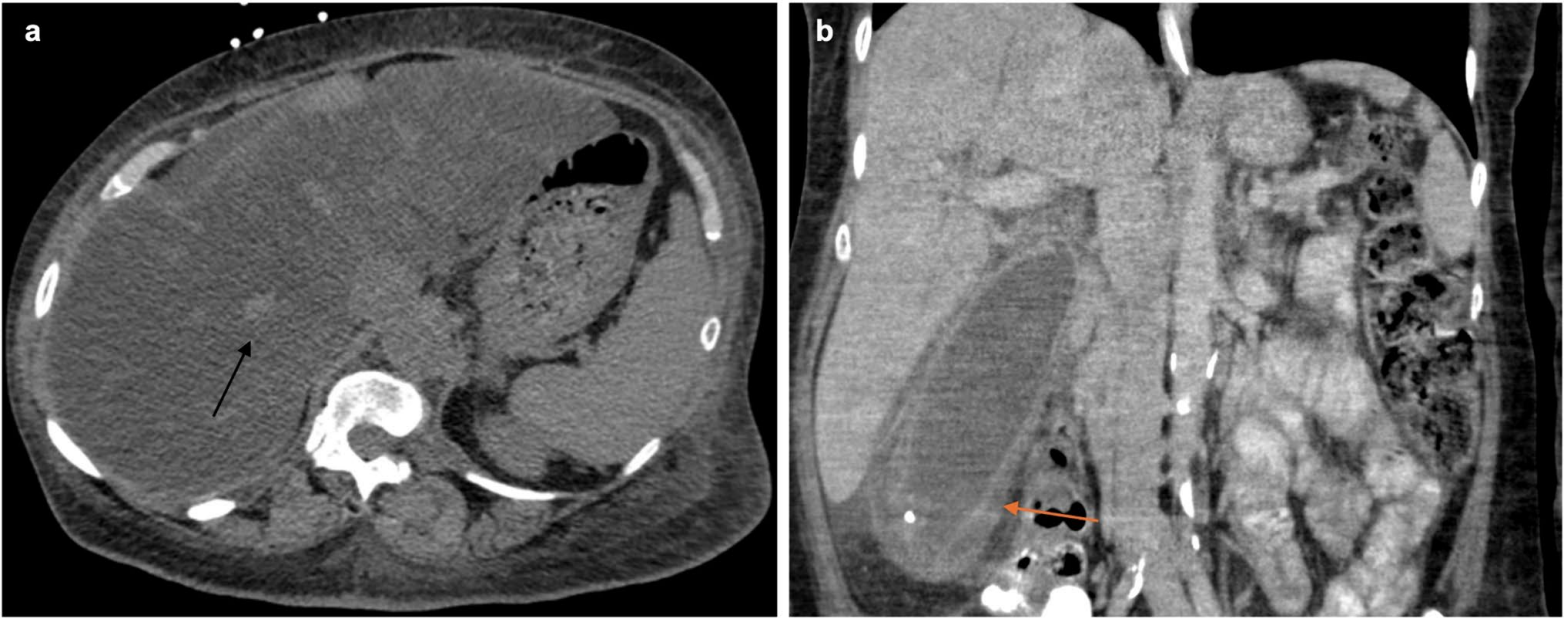
Findings associated with toxicity from usage of checkpoint inhibitors. CT abdomen (**a**) demonstrating hepatitis (black arrow) with hepatomegaly, heterogeneous hepatic contrast enhancement, well-defined parenchymal zones with low attenuation, and periportal hypoattenuation. Coronal CT (**b**) demonstrates gallbladder distention, mucosal hyperemia, wall thickening, pericholecystic fat stranding/fluid consistent with cholecystitis (orange arrow)

The liver, with its hypervascularity and metabolic function, is frequently affected by either GE cancer metastases or chemotherapy-related toxicity [37]. Given the limited sensitivity of symptom-based approaches, the National Cancer Institute introduced a comprehensive set of criteria to identify chemotherapy-associated adverse effects, including clinical status, laboratory results, and imaging findings [37]. With the advent of novel therapies, imaging has emerged as an effective and sensitive tool for detecting side effects in patients with advanced GE cancer. For example, it has been shown that fluoropyrimidine-toxicity can present as hepatic steatosis or biliary strictures, seen as a hyperechoic liver on ultrasound or as an obstructive appearance on MRCP (Fig. 8) [38]. Similarly, platinum-based chemotherapy can cause hepatomegaly, ascites, gallbladder edema, wall thickening, and periportal edema (Fig. 8) [39]. Finally, various immunotherapeutic agents have been associated with hepatitis and cholecystitis, including nivolumab, pembrolizumab, ipilimumab, trastuzumab, and ramucirumab (Fig. 9) [40].

### Gastrointestinal sequelae

Therapy-associated gastrointestinal (GI) symptoms, such as nausea, vomiting, and diarrhea, are the most common and most associated adverse effects with decreased quality of life in cancer patients [41]. While these symptoms may sound benign, their severity can result in discontinuation of therapy, having a significant impact on treatment efficacy and survival [42]. One of the most common presentations concerning medication-related toxicity of the gastrointestinal system is colitis, with a reported incidence of 2% in patients using mono-immunotherapy and 12% in patients undergoing combined regimens [43]. Immunotherapy-induced colitis can non-invasively be diagnosed with ultrasound or CT imaging, though definitive diagnosis requires endoscopic evaluation with biopsies. Endoscopically, the pattern of inflammation is heterogeneous without a single defined appearance [43]. Other signs of adverse reactions to treatment include pancreatitis and, more dramatically, GI perforation [44]. Major studies evaluating bevacizumab in combination with 5–FU–based regimens such as IFL, 5-FU–LV, and FOLFOX have revealed GI perforation rates of 0% to 3.3% [43, 44].

**Fig. 10:**
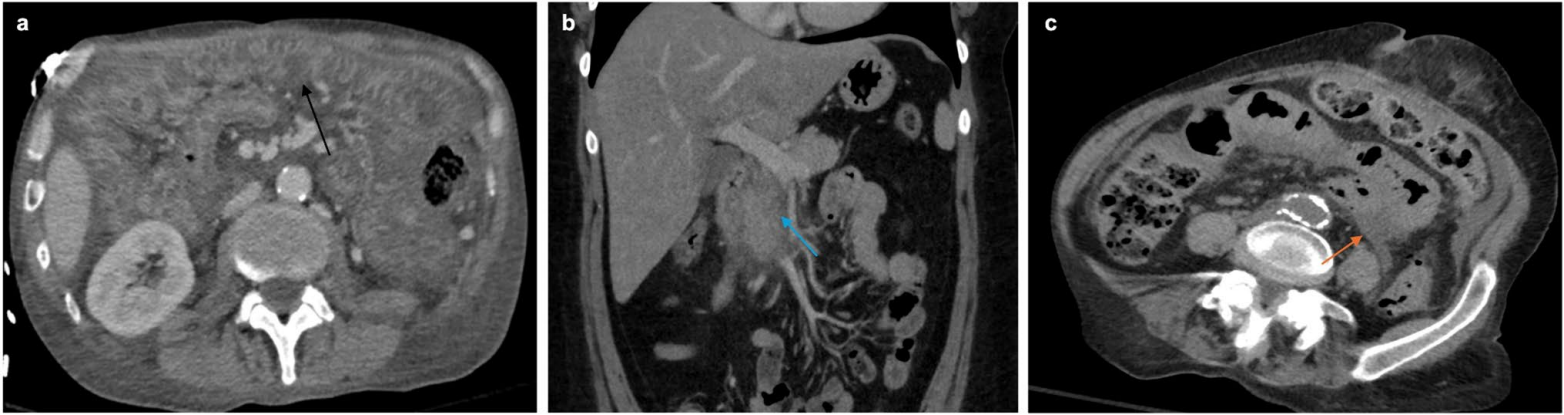
CT abdomen/pelvis axial view (**a**) demonstrates severe, diffuse treatment-associated colitis (black arrow), recognizable by nodular indentations of the lumen (aka thumbprinting), mucosal hyperemia, extensive fat stranding, wall thickening, and a pattern known as the accordion sign. Coronal CT abdomen (**b**) and axial CT (**c**) reveal pancreatitis (blue arrow) recognized by a markedly enlarged and irregular-appearing pancreas with extensive infiltration of peripancreatic fat in addition to peripancreatic fluid (orange arrow)

Immunotherapy-related colitis can appear on CT as one of two different types: diffuse or segmental. Segmental, or focal, colitis is seen when a short section of the bowel has increased wall thickness with mucosal enhancement and pericolic fat standing [38]. In contrast, diffuse colitis, as characterized by CT, is when a long segment of the colon demonstrates wall thickening, mucosal enhancement, engorgement of mesenteric vessels or fluid in the bowel lumen [38]. Additional CT findings of colitis-related perforation include bowel wall discontinuity, extraluminal air, and abrupt bowel wall thickening with or without an associated phlegmon (Fig. 10).

## Radiation side effects

### Pulmonary findings

Radiation is crucial in treating advanced esophageal cancer but requires careful evaluation for potential side effects. Pulmonary toxicity should be monitored closely in patients undergoing radiation, as research shows that the lungs are particularly vulnerable to radiation-related damage [39]. The toxicity can be classified by time of onset: early radiation-induced organizing pneumonia (RIOP) and late chronic radiation-induced lung fibrosis (RILF). Symptoms in recently initiated patients such as coughing, shortness of breath, chest tightness, and signs of respiratory distress may indicate the development of radiation-induced organizing pneumonia [39, 40]. RIOP is different from the cryptogenic organizing pneumonia seen with medical therapies due to its presence outside the irradiated area and its occasional migratory nature. Notably, RIOP also carries a higher mortality rate compared to COP [39, 40]. If the damage to the lung becomes severe, RIOP might lead to fibrosis and, ultimately, respiratory failure. Extensive RILF can present as progressive dyspnea, pulmonary hypertension, and cor pulmonale [45].

**Fig. 11:**
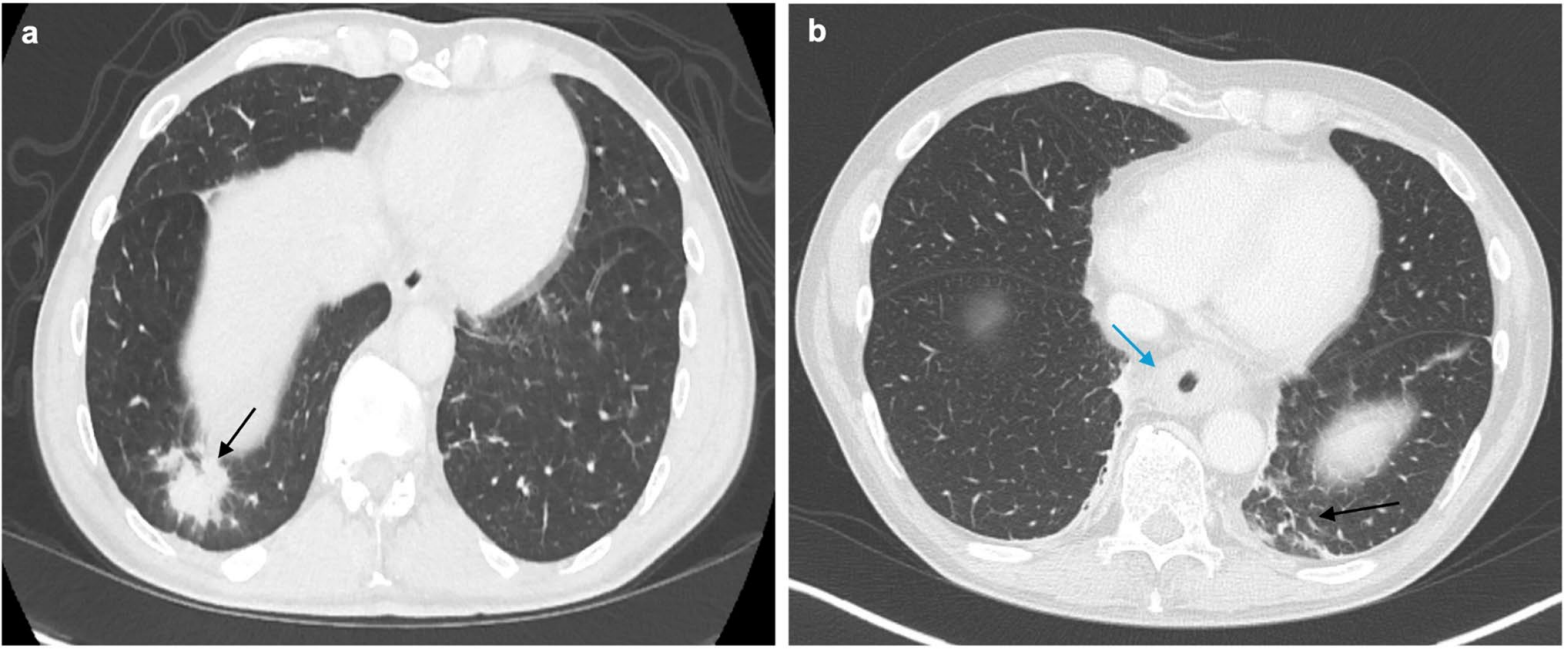
CT scan of the chest (**a**,** b**) demonstrates patchy consolidative opacities with ground-glass attenuation and fibrosis (black arrows) which are characteristic findings of radiation-related organizing pneumonia. There is also radiation-induced wall thickening of the esophagus (blue arrow)

Immediately following radiation therapy, chest radiographs typically display focal or multifocal opacification in the lung fields, which may be detected without the need for CT scans. If performed, CT scans at this phase reveal ground-glass and reticular opacities that progress to scar-like consolidations and traction bronchiectasis (Fig. 11) [39, 40, 45]. These changes may resolve within weeks, or over the subsequent 6–9 months, noticeable areas of fibrosis may develop. Late fibrosis appears radiologically as sharply defined consolidative or linear scarring with volume loss and architectural distortion, sometimes accompanied by septal wall thickening, causing a “crazy paving” pattern [45].

### Cardiac toxicities

Radiation-induced cardiac dysfunction can occur months to year following radiation exposure. Most of the work done to validate this claim was done in patients with breast cancer and Hodgkin’s lymphoma, in home the following sequelae were seen: arrhythmias, cardiomyopathy, valvular abnormalities, ischemic heart disease, and pericarditis [46, 47]. Other factors, such as smoking and hypertension, can contribute to the development of these treatment-related complications [48]. The imaging techniques utilized to assess for radiation-induced cardiotoxicity are the same as those used to investigate the adverse effects of chemotherapy. On echocardiography, radiotherapy-related heart valve damage presents as fibrosis and calcification in the aortic root, aortic valve, mitral valve, and parts of the mitral valve leaflets [48]. Likewise, CMR may reveal fibrosis, edema, and generic tissue damage like pericardial thickening or pericardial effusion [48, 49]. Tissue destruction will present with an increased T1 signal, while edema will show increased T2 [49]. Recent work in animal models claims that myocardial strain on CMR is a sensitive imaging biomarker for detecting radiotherapy-induced subclinical systolic or diastolic dysfunction before the complete deterioration of global cardiac function [49].

### Esophageal complications

**Fig. 12:**
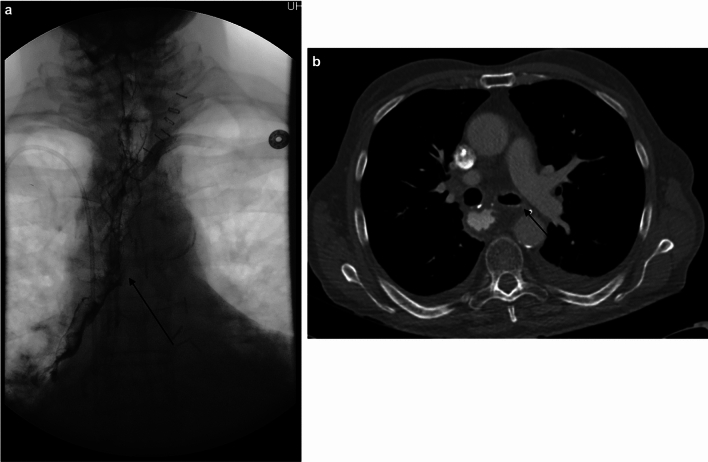
Esophagogram (**a**) demonstrates a tracheoesophageal fistula (arrow) with contrast opacification into the bilateral bronchial tree. Axial CT (**b**) shows contrast pooling in the esophagus with small amounts of contrast in the dependent aspect of the left mainstem bronchus (arrow)

Another common consequence of radiation treatment is esophagitis, characterized by inflammation, edema, and erosion of the esophageal mucosa. The underlying pathophysiology involves the production of free-radical oxygen species by cytokines released during radiation-induced cell death [50]. Higher radiation doses and concurrent chemotherapy increase the incidence of radiation esophagitis, leading to symptoms such as difficult or painful swallowing and secondary malnutrition [50]. Esophagitis can be classified as acute if it develops within 3 months after therapy or late if it occurs more than 3 months after treatment. Similarly, a CT of the chest and abdomen can also assess fistulization, esophageal wall thickening, and surrounding inflammatory changes (Fig. 12). Upper endoscopy remains the gold standard because it allows direct visualization of the mucosa and facilitates biopsy, though complex cases may benefit from a multimodal approach. For instance, some patients may have a normal esophagram due to superficial mucosal involvement in acute radiation-associated esophagitis. In that case, CT can be performed and may reveal focal esophageal wall thickening along the irradiated esophagus, the extent of which depends on the chronicity of the symptoms [51]. Moreover, other acute cases may benefit first from fluoroscopic studies to uncover findings like abnormal peristalsis and serrated contour from wall edema [51].

### Myelosuppression

Radiation-induced myelosuppression refers to a functionally subdued bone marrow that produces fewer platelets, erythrocytes, and leukocytes, creating obvious hematologic consequences. [52, 53]. This is a common side effect of radiotherapy that is dose-dependent and highly related to the lifetime total equivalent radiation received [52, 53]. Guidelines recommend interventions such as pegylated recombinant human granulocyte colony-stimulating factor (G-CSF) for patients with significantly low leukocyte or platelet counts [53]. Recommended imaging modalities for assessing radiation-induced myelosuppression include bone marrow imaging with MRI or PET-CT, which can provide quantitative information on bone marrow cellularity [52, 53]. In the first 24–72 h following radiation, MRI of the bone marrow is significant for hypointense signal on T1-weighted images and hyperintensity on fat-saturated T2-weighted images, indicating the presence of edema. Subsequently, focal areas of bleeding may occur [52–54]. The bone marrow ultimately undergoes a conversion to fatty marrow in a manner that closely reflects the physical field of radiation and which appears bright on T1-weighted sequences and dark on fat-suppressed images [54]. Eventually, a bone marrow biopsy may also be necessary for a definitive diagnosis.

## Surgical complications

### Gastric conduit dysfunction

Functional issues can arise following esophagectomy, like delayed gastric emptying, reflux, dumping syndrome, and difficulty swallowing [55]. These problems can contribute to weight loss after Ivor–Lewis esophagectomy and may be associated with lower long-term survival rates [56]. Delayed gastric emptying is the most frequent issue, affecting nearly 50% of patients [56]. Diagnosis involves clinical symptoms confirmed by an upper gastrointestinal fluoroscopic study or a nuclear medicine gastric emptying study but might not fully evaluate fluid versus solid gastric emptying. If left untreated, this could result in a redundant gastric conduit, necessitating further surgical intervention. Findings that might indicate conduit dysfunction on imaging are dilated gastric conduit, retained food or fluid within the conduit, delayed emptying of contrast material, and abnormal peristalsis or motility is seen on fluoroscopy or dynamic CT imaging [55, 56].

### Gastric conduit ischemia or necrosis

The literature suggests that gastric conduit ischemia should be classified as something distinct from anastomotic leak. Conduit necrosis is a rare but severe complication following esophageal surgery with a mortality rate as high as 90% but an incidence of only 1–3% [56]. Because of its infrequent but dreaded presentation, there should be a low threshold to image postoperative patients with symptoms such as pain, fever, tachycardia, and sepsis without evident cause [56, 57]. CT plays a vital role in diagnosing gastric conduit ischemia or necrosis, displaying findings such as thickening of the gastric wall, decreased mucosal enhancement of the gastric conduit, pneumatosis, and peri-conduit fluid collection [57]. Treatment for conduit ischemia begins with supportive care and antibiotics. Still, severe cases require emergency debridement, exteriorization of the cervical esophagus, and repositioning of the residual vital gastric conduit back to the peritoneal cavity [57].

### Anastomotic leakage

Esophagectomy with gastric pull-up can be performed in end-stage benign esophageal disease or cases of esophageal rupture, but it is mainly performed to treat esophageal cancer. For this purpose, numerous surgical techniques have been described, with transthoracic (Ivor Lewis, McKeown, thoracoabdominal approaches), transhiatal, and minimally invasive esophagectomies being the most common [58]. With an incidence between 6 and 28%, anastomotic leak is a frightening complication of esophagectomy that prolongs hospitalization and increases mortality and cost [58]. The clinical presentations of anastomotic leakage post-esophagectomy are quite severe and can be fatal. Respiratory compromise and unstable hemodynamics can arise if the gastroesophageal anastomosis leak contaminates the pleural, leading to infection and obstruction that affect intrathoracic pressure [59]. Yet, because of its high sensitivity and specificity, endoscopy has become the most reliable method to quickly identify anastomotic leaks, to the point that some institutions protocol that patients undergo an esophagram postoperatively to ensure safe disposition [58, 59].

### Paraconduit hiatal hernia

Paraconduit hiatal hernia (PHH) is a known complication of esophagectomy and is associated with younger age and tumor recurrence [55]. As with other hernias, the significant risks in PHH are bowel strangulation and perforation. The incidence of PHH varies depending on diagnostic criteria. One survey found that 1.18% of post-esophagectomy patients required additional surgical interventions due to PHH, with a notable portion needing emergency surgery due to critical complications such as hemodynamic instability [55]. It is noteworthy that laparoscopic introduction to esophagectomy has been linked as a risk factor for PHH [60]. Early diagnosis and intervention are crucial given the potential acute complications within the first year post-esophagectomy, even in asymptomatic patients [55]. While endoscopy remains the gold standard for evaluating anatomical abnormalities within the esophagus, PHH is more often incidentally diagnosed via CT [55, 60]. Findings will usually show loops of bowel and fat herniating into the thoracic cavity, causing atelectasis of adjacent lung parenchyma, displacement of other organs such as the colon or small bowel, and signs of obstruction [60].

## Conclusion

The rapidly evolving management of gastroesophageal cancer relies on the collaborative expertise of a large multidisciplinary team, including pathologists, medical oncologists, radiation oncologists, surgical oncologists, and radiologists. To effectively contribute to this team, radiologists need a foundational understanding of gastroesophageal cancer and the questions that guide clinical decision-making. The recent update to the ASCO treatment guidelines for gastroesophageal cancer in 2023 represents a monumental step—a shift of the field towards precision oncology. The ASCO guidelines provide a high-yield framework that radiologists can leverage to highlight the current treatment guidelines, their unintended consequences, and their corresponding radiographic findings. Only through a comprehensive approach of bridging pathology, pharmacology, radiation, and surgery can radiologists demonstrate their indispensable value in the multidisciplinary cancer team.
